# Identification and analysis of mtDNA genomes attributed to Finns reveal long-stagnant demographic trends obscured in the total diversity

**DOI:** 10.1038/s41598-017-05673-7

**Published:** 2017-07-21

**Authors:** Sanni Översti, Päivi Onkamo, Monika Stoljarova, Bruce Budowle, Antti Sajantila, Jukka U. Palo

**Affiliations:** 1 Department of Biosciences, University of Helsinki, PO Box 56, FI-00014 Helsinki, Finland; 2 Department of Gene Technology, Tallinn University of Technology, Tallinn, Estonia; 3 Center for Human Identification, University of North Texas Health Science Center, 3500 Camp Bowie Boulevard, Fort Worth, Texas USA; 4 Center of Excellence in Genomic Medicine Research (CEGMR), King Abdulaziz University, Jeddah, Saudi Arabia; 5 Department of Forensic Medicine, University of Helsinki, PO Box 40, FI-00014 Helsinki, Finland; 6 Forensic Genetics Unit, National Institute for Health and Welfare, PO Box 30, FI-00271 Helsinki, Finland

## Abstract

In Europe, modern mitochondrial diversity is relatively homogeneous and suggests an ubiquitous rapid population growth since the Neolithic revolution. Similar patterns also have been observed in mitochondrial control region data in Finland, which contrasts with the distinctive autosomal and Y-chromosomal diversity among Finns. A different picture emerges from the 843 whole mitochondrial genomes from modern Finns analyzed here. Up to one third of the subhaplogroups can be considered as Finn-characteristic, i.e. rather common in Finland but virtually absent or rare elsewhere in Europe. Bayesian phylogenetic analyses suggest that most of these attributed Finnish lineages date back to around 3,000–5,000 years, coinciding with the arrival of Corded Ware culture and agriculture into Finland. Bayesian estimation of past effective population sizes reveals two differing demographic histories: 1) the ‘local’ Finnish mtDNA haplotypes yielding small and dwindling size estimates for most of the past; and 2) the ‘immigrant’ haplotypes showing growth typical of most European populations. The results based on the local diversity are more in line with that known about Finns from other studies, e.g., Y-chromosome analyses and archaeology findings. The mitochondrial gene pool thus may contain signals of local population history that cannot be readily deduced from the total diversity.

## Introduction

The current genetic diversity of any population is a result from the actions of four evolutionary forces (drift, mutation, migration and selection). Reconstructing the past from modern genetic data can thus be challenging. The confounding signals resulting from these different forces can be minimized to some extent, e.g., by marker choice (ignoring selection) or by assuming steady mutation rates, but genetic drift and gene flow are more difficult to address. This issue is especially problematic in human populations due to relatively high migration rates and very short genetic distances between populations with evolutionarily young species age and small founding populations. Yet, the two latter forces are the most important ones when inferring population history as they reflect changes in population size and migration patterns during different phases of history.

An increasingly popular approach to elucidate different demographic events is to analyze past diversity directly from archaeological samples, i.e., ancient DNA analyses. Despite recent technical and analytical advances this approach is still relatively expensive, time-consuming, error-prone and often involves very limited size data sets. An alternate approach is to use modern genetic data and indirect means to deduce the past. These studies could include simulation methods that allow evaluation of the likelihoods of alternative demographical scenarios or *a priori* selection of sampled individuals based on, e.g., locality or surnames (for review see ref. 1). In the study herein the rationale followed was exploring demographic signals in two different spatiotemporal layers of mitochondrial diversity by segregating the modern data into “local” and “immigrant” components^2^. This approach revealed demographic history patterns that remained hidden within the total population variation.

The population of Finland was explored because of its rather well-studied, and peculiar history. This history is branded by distinct colonization waves, high drift due to small size and isolation-by-distance^3, 4^. Y-chromosomal and genome wide studies support that Finnish genetic diversity patterns differ compared with most other West Eurasian populations (see e.g. ref. 5). The Y chromosomal diversity is especially reduced and distinctive, showing a clear east-west divergence over short geographic distances within the country^4–8^. This genetic difference may have arisen from an ancient border between two different immigration waves that arrived at different times and could represent two different modes of subsistence, i.e., hunter-gathering (east) and animal husbandry/farming (west)^9^. A similar but less dramatic border has been observed with the autosomal data^8^ and even in genes with pharmacogenetic relevance^10, 11^.

Whereas the reduced and distinctive Y-chromosomal diversity suggests a history of population bottlenecks and immigration waves, most mtDNA studies have observed an overall pattern similar to that of most other European populations, i.e. high diversity and low geographical segregation of diversity^4, 6, 9, 12–14^. Finns carry mostly European haplogroups H, I, J, K, T, V, W and X, but also to some extent Asian haplogroups D and Z^12^. However, some studies have observed weak geographical patterns in the Finnish mtDNA diversity^4, 9, 13^. The Finns show vestiges of an ancient border between the Mesolithic hunter-gatherer (U and V, see Discussion) and Neolithic farmer mtDNA haplogroups (H, J, K and T), a pattern previously identified in their Y-chromosomes^9^.

The demographic signals in the two notable components of mtDNA variation, each with different histories in Finland, were studied. Based on the geographical distribution, mtDNA lineages were searched in public repositories. Unlike earlier studies focusing on Finland, this study aimed to achieve a fine resolution picture by analyzing only full mtDNA genome sequence data. A significant proportion of subhaplogroups in a Finnish data set, up to one third, are rare or absent outside Finland, a mtDNA component henceforth called “Finn-characteristic” lineages. The results indicate that separate analyses of local and immigrating lineages allowed detection of different population history signals. In this study, this signal was most evident in pre-historic effective population sizes. Most importantly, these patterns have not been detected within total population variation studies.

## Material and Methods

### Database searches and haplogroup assignment

Complete modern mtDNA genomes from Finland were obtained through GenBank searches, HmtDB searches^15, 16^ and from published literature, including information on the population of origin. Altogether 843 complete Finnish sequences were retrieved (Table 1 and Supplementary Dataset File). Sequences were aligned with Muscle v3.8.31^17^ to the revised Cambridge reference sequence, rCRS^18^. Finnish samples used in this study might be slightly biased towards the northern parts of Finland: dataset contained samples from northern Ostrobothnia, central Ostrobothnia, Kainuu and northern Savo in refs 19 and 20 and Northern Finland in ref. 21. Nevertheless, the samples from Tampere^22^ can be considered more representative of southern and western parts of the country. More detailed place of origin was not available for all samples^23, 24^.

**Table 1:** Modern human complete mtDNA genomes from Finland (N = 843).

N	Reference	GenBank ID numbers	Origin
93	24	n/a	n/a
192	19	AY339402–AY339593	Northern Ostrobothnia, central Ostrobothnia, Kainuu, and northern Savo
94	23	Supplementary Table S1	n/a
293	22	Supplementary Table S1	Tampere
64	21	JX171077–JX171140	Northern Finland
79	20	KC763372–KC763450	Northern Ostrobothnia
28	*,**^43^, ^68^	AY195779*, JF837819**, EU753433, EU784076, FJ543390, FJ801039, GQ176284, GU206811, GU391321, GU949563, HM116534, HM856585, HQ022823, HQ658464, HQ840516, JF298814, JF813785, JF813786, JF903928, JQ086344, KF466256, KF631316, KM213522, KP782041, KR779775, KR712271, KR919601, KR902539	n/a
**TOTAL 843**			

Sequences were obtained through GenBank searches, 1000 Genomes data and from previously published articles.

It is worth noting that altogether 335 of the Finnish sequences (published in refs 19–21) were obtained by sequencing mtDNA genomes initially showing variation by conformation-sensitive gel electrophoresis (CSGE). The potential influence of this approach on the results is discussed below (see Results).

The mtDNA haplogroup information for all sequences was inferred from the sequence data both by using Haplofind^25^ and HaploGrep2^26^, in order to gain greater certainty for the haplogroup definitions. Haplofind is based on PhyloTree version 16^27^ HaploGrep2 is based on PhyloTree version 17^27^. For less clear haplogroup assignments the results from HaploGrep2 were considered to be more reliable and relied upon in this study.

To identify mitochondrial lineages characteristic for Finns BLAST (Basic Local Alignment Tool^28^) searches were conducted against sequences deposited in GenBank to determine the geographical distribution of haplotypes in the different (sub)haplogroups. The GenBank database contains around 30,000 complete human mitochondrial genomes (estimated from GenBank http://www.ncbi.nlm.nih.gov/genbank/ and Human Mitochondrial database http://www.hmtdb.uniba.it/hmdb/ 15.03.2017). This resource thus is a larger database than that of the commonly used PhyloTree database (24,275 complete or nearly complete genomes; 15.3.2017). PhyloTree and GenBank databases overlap, with over 90% of complete genomes deposited in PhyloTree also housed in GenBank.

As a query sequence for each BLAST search one complete mitochondrial sequence from each possible Finnish characteristic haplogroup was used. For each search the maximum number of sequences to display was set to 500 and for all these 500 sequences the haplogroup was determined by HaploGrep2. Haplogroups, which contained less than four sequences in our Finnish sample (such as H1f1a, H1a8 and U5b2a1a1b) or which were known to be common in other populations (e.g. H2a1, U5b1b1a and V7a1), were excluded from the searches. The ethnic origins of the sequences were inferred from GenBank and/or from original publications. Estimated from the Human Mitochondrial DataBase, around 20% of complete mtDNA sequences in GenBank are of undefined origin (Supplementary Table S1). Note, however, that roughly one fifth of the GenBank sequences lack precise information on ethnic/geographical origin and the origin for those sequences is designated as “n/a” (see Results). Despite the uncertainty of the ethnic/geographical origin of these sequences, all sequences deposited in GenBank were included in the subsequent analysis to avoid an overestimation of the proportion of Finn-characteristic haplogroups. It is possible that these sequences also include Finnish samples, which in turn would indicate that the Finn-characteristic haplogroups could be even more restricted to Finns than it appears based on the results. The numbers of complete mtDNA sequences from nearby populations are presented in Supplementary Table S2.

Haplogroups, for which Finns constituted more than 75% of the sequences, were considered Finn-characteristic. In order to visualize the diversity within these haplogroups, Neighbor-Joining (NJ) trees were reconstructed with MEGA 6^29^ using substitution model TrN+ Γ with shape parameter *α* = 0.02. The choice of the model was based on the best-fit model estimated with PartitionFinder^30^ (TrN+ Γ+ Inv) and gamma shape parameter estimated by BEAST v1.8.2. Minor modifications of these values were necessary due to MEGA software limitations. Trees were rooted with L3a haplogroup sequence JN655813^31^. The branch-support values were obtained by bootstrapping (500 resamples). NJ trees are presented in the Supplementary Figs S1–S4.

As the 75% inclusion limit is arbitrary, it was necessary to assess whether the obtained results are heavily dependent on the cut-off limit set to define Finn-characteristic haplogroups. A more stringent cut-off limit of 90% also was applied to the data, and the results obtained with the different data sets were compared.

### Phylogenetic analyses

In order to estimate the age of the haplogroups and past population sizes the Bayesian approach in BEAST v1.8.2^32^ was used. For these analyses, the optimal partition scheme of the mtDNA variation and the best-fit nucleotide substitution model for each partition were estimated using the software PartitionFinder^30^ assuming seven categories of nucleotides:

1. Nucleotides 1–576 (including hypervariable region 2)
2. Nucleotides 16,024–16,569 (including hypervariable region 1)
3. Protein coding positions at 1^st^ codon (PC1)
4. Protein coding positions at 2^nd^ codon (PC2)
5. Protein coding positions at 3^rd^ codon (PC3)
6. Transfer RNAs (tRNAs)
7. Ribosomal RNAs (rRNAs)

PartitionFinder analyzes every scheme that includes these seven categories in all possible combinations. The best partitioning was obtained for four schemes (1 + 2, 3, 4 + 5 and 6 + 7). The most appropriate nucleotide substitution model for each scheme is presented in Table 2. The preferred model, the General Time Reversible model^33^, was too complex of a model for scheme 1 (non-coding control region nucleotide positions 1–576 and 16,024–16,569) with the subsequent Bayesian phylogenetic analyses. Therefore, the GTR model was changed into the more simplified Tamura-Nei model^34^ and was used in all subsequent analyses (Table 2).

**Table 2:** The best-fit nucleotide substitution model and substitution rate estimation for each partitioning schemes.

Scheme number	Composition	Best model according to the PartitionFinder	Best model according to the Bayesian analysis with BEAST v1.8.2	Substitution rate this study (μ/Site/Year × 10^−8^)	Substitution rate Rieux et al. 2014 (μ/Site/Year × 10^−8^)
**1**	Nucleotides 1–576 and 16,024–16,569	GTR+ Γ+ Inv	TrN+ Γ+ Inv	20.56 [12.49, 29.01]	31.43 [22.56, 40.31]*
**2**	rRNA, tRNA	HKY+ Γ+ Inv	HKY+ Γ+ Inv	1.58 [0.94, 2.30]	1.01 [0.76, 1.27]
**3**	PC1, PC2	TrN+ Γ+ Inv	TrN+ Γ+ Inv	1.80 [1.17, 2.40]	0.76 [0.57, 0.94]
**4**	PC3	TrN+ Γ+ Inv	TrN+ Γ+ Inv	3.04 [1.92, 4.21]	3.32 [2.57, 4.07]

GTR = General Time Reversible^33^, HKY = Hasegawa-Kishino-Yano^69^, TrN = Tamura-Nei^34^, Γ = Gamma distribution and Inv = Invariant sites. In all the models the number of gamma categories was set to 4. Substitution rates are the mean with 95% highest posterior density interval. For the comparison, the substitutions rates obtained from Rieux *et al*.^38^ also are presented.

*Mutation rate estimated for hypervariable region 1 and 2 (HVR1 + HVR2).

All the Bayesian phylogenetic analyses were performed in BEAST v1.8.2^32^. Each BEAST run was performed in the same way: single MCMC chain was ran for 40,000,000 steps, sampled every 4,000 steps and the first 10% was discarded as a burn-in. For each of the following analyses four independent runs were performed, and these runs were combined with LogCombiner to achieve adequate effective sample size (ESS) values^35^. Appropriate effective sample size values (ESS > 200) for each parameter in the model were checked in Tracer v1.6^36^.

Substitution models and clock models were unlinked between the schemes in all analyses; but the tree topology was assumed to be the same between the four schemes. The most suitable demographic and clock models were determined by Bayes factors calculated from the marginal likelihoods^37^. The data were fitted into three demographic models (Bayesian skyline plot, exponential growth and constant population size models). The most appropriate demographic model based on Log_10_ BF comparison was the constant population size model (Log_10_ BF 1.92 and 2.22 compared to the BSP and exponential growth models, respectively). The constant population size model is considered to be most suitable for population level analyses (see http://www.beast2.org/tree-priors-and-dating/ 15.03.2017). The most suitable molecular clock for the data was strict clock (Log_10_ BF 4.05 over lognormal relaxed clock). The prior distributions for mutation rates for each of the four partitions were set based on Rieux *et al*. 2014^38^.

Because the tip calibration has been shown to yield more consistent results than node dating^38^, 12 previously published radiocarbon dated sequences were used as calibration points in our analyses (Supplementary Table S3). The tip date uncertainty was taken into account, as suggested by Rieux *et al*. 2014^38^. The 12 ancient sequences were from different time points as it has been shown that even a small sample of aDNA sequences with widely distributed and accurate ages are sufficiently powerful to produce precise estimates for mutation rate and timescales^39^.

### Bayesian Skyline Plots

The effective population sizes for Finn-characteristic and non-Finn-characteristic haplogroups were compared by constructing Bayesian Skyline Plots (BSPs)^40^ with BEAST v1.8.2^32^. The coding region (nucleotides 577–16,023) was used in BSP analyses. The best-fit substitution model was estimated with PartitionFinder^30^. The substitution model used was TrN+ Γ+ Inv with four gamma categories and partitioned into codon positions (1 + 2), 3. The tree prior used was piecewise-linear Skyline coalescent model with number of groups 10. The prior normal distribution for mutation rate was estimated based on six previously published mutation rates for mtDNA coding region^41–46^. Resulting mean mutation rate was *μ* = 1.546 × 10^−8^ ± 3.675 × 10^−9^ (SD) substitutions per base pair per year.

In order to test the robustness of analysis, BSPs were constructed for those Finn-characteristic haplogroups for which Finns constituted ≥90% of the sequences.

As an additional test for validity of obtained results BSP plots were constructed using HVR1 + 2 data (643 bp) from Neuvonen *et al*. 2015^9^, of which geographical origin information was available. In order to explore potential population structure effects, the BSPs were constructed separately for eastern Finland, western Finland and all data (see ref. 9) assuming the same run parameters as for the 1 + 2 partition scheme described above.

## Results

### Finnish mtDNA diversity

A total of 843 Finnish mitochondrial genomes were retrieved from the databases. These data segregate into 240 subhaplogroups (Supplementary Table S4) with overall haplogroup frequencies similar with those published previously^9, 12, 13^. To evaluate a possible sampling bias, due to lack of precise information of the geographical origin of the Finnish samples used in this study, the main haplogroup frequencies were compared between this study and HVR1 + 2 data presented in Palo *et al*. 2009^4^. In Palo *et al*. 2009 the donor’s reported place of residence was known, and the sampling covered Finland as a whole. There were no significant differences in the frequencies between these two studies, which supports that the data of this study can be considered a reasonable representative sample of Finland. (Supplementary Table S5).

Unlike most of the sequences, a significant portion of the Finnish sequences (n = 335) deposited in GenBank were originally obtained by sequencing only representative mtDNA genomes that showed variation with CSGE. The reliability of these data therefore depends on the capability of the CSGE technique in detecting variation among genomes. This was not considered a significant source of bias. First, as with the sampling bias discussed above, congruence in haplogroup frequencies between this study and previous ones supports data coherence. Second, the CSGE method has shown to be able to resolve single base pair differences in Körkkö *et al*. 1998^47^.

Using the cut-off limit of 75% (Table 3), a total of 281 Finn-characteristic sequences belonging to 23 mitochondrial lineages was identified. These lineages constituted up to 33.3% of our whole data set and comprised both hunter-gatherer (haplogroups U and V) and farmer (haplogroups H, J and K) associated lineages^48^. With the more stringent cut-off limit of 90%, 20.8% of all mtDNAs were designated Finn-characteristic. The proportion of Finn-characteristic haplogroups within each dataset used^19–24^ varied between 20.2–45.3% (Supplementary Table S6).

**Table 3:** Finn-characteristic haplogroups.

Haplogroup	N *	N Finn/% **	Others (GenBank ID, origin if available)	Prevalence in Finland based on this study (N_Finns_ = 843)
H13a1a1d1	11	11/100.0	—	1.3
H1a2	26	21/80.8	EU687746.1 Saami Norway, HM775496.1 Sweden, EU130562.1 n/a, JQ702405.1 n/a, EST_27	2.5
H1f1	30	27/90.0	JQ704916.1 n/a, JQ705985.1 n/a, EST_15	3.2
H1n4	13	10/76.9	JQ702043.1 n/a, EST_45, EST_75	1.2
H3h1	17	14/82.4	KF162052 Denmark, KJ446376 = KF451393 Russia, EST_5	1.7
H5a1e	4	4/100.0	—	0.5
I1a1a1	5	5/100.0	—	0.6
I1a1a2	4	4/100.0	—	0.5
I2b	4	4/100.0	—	0.5
J1c2n1	8	8/100.0	—	0.9
K1c1c	22	18/81.8	KC170989 Kirov Russia, KF162649 Denmark, EU262720 n/a, EST_1	2.1
U5a2a1a	14	13/92.9	KF161330 Denmark	1.5
U5b1b1a1a	11	9/81.8	GU296598.1 Russia, JQ702500.1 n/a	1.1
U5b1b1a1a1	7	7/100.0	-	0.8
U5b1b2	30	23/76.7	JX026063.1 Sweden, JQ702152.1 Ireland, KF161244.1 Denmark, KF162959.1 Denmark, KF162429.1 Denmark, EU367993.1 n/a, EST_18	2.7
U5b1b2a	12	11/91.7	EST_107	1.4
V1a1a	6	6/100.0	—	0.7
V1a1a1	12	12/100.0	—	1.4
V5	10	9/90.0	JQ705942.1 n/a	0.7
V8	14	11/78.6	JQ705604.1 n/a, JQ703985.1 n/a, JQ702025.1 n/a	1.3
W1a	37	35/94.6	KF061034 England, EST_37	4.2
W1b	4	4/100.0	—	0.5
W1b1	14	14/100.0	—	1.7
Total	315	281/89.2	—	33.3

Estonian samples (EST_27, EST_15, EST_45, EST_75, EST_5, EST_1, EST_18, EST_107 and EST_37) from Stoljarova *et al*.^70^. n/a = ethnic/geographical origin for the sequence not available.

*Number of sequences belonging to the haplogroup.

**Number of Finnish sequences belonging to the haplogroup/the percentage of Finnish sequences in the haplogroup.

Based on the Bayesian phylogenetic analysis the mutation rates obtained in this study were similar with rates previously published by Rieux *et al*. 2014^38^ (Table 2). Slight differences were observed: the mutation rate for PC1 + PC2 was slightly higher (*μ* = 0.76 × 10^−8^ mutations/site/year in Rieux *et al*. 2014 vs. *μ* = 1.80 × 10^−8^ mutations/site/year in this study) and the rate for nucleotides 1–576 and 16,024–16,569 (HVS1 + HVS2) was slightly lower compared to Rieux *et al*. 2014 rates. These differences are minor and probably arise from the different data sets used in these studies. For each of the schemes the base frequencies, transition/transversion ratios, proportion of the invariant sites and the shape parameter *α* were estimated from the data (Supplementary Table S7). In the control region the transitional rate between pyrimidines has been shown to be higher than that between purines^34^. For protein-coding genes the mutation rate for guanine has been estimated to be very high compared to other parts of the mitochondrial genome^49^ resulting in higher transitional rate between purines. The same phenomena were visible in these data. The gamma distribution shape parameter *α* was the highest in the scheme 1 (nucleotides 1–576 and 16,024–16,569 with *α* = 0.62 ± 0.29), and the proportion of invariants was the highest in scheme 4 (rRNAs and tRNAs, pInv = 0.94 ± 0.03).

**Figure 1:**
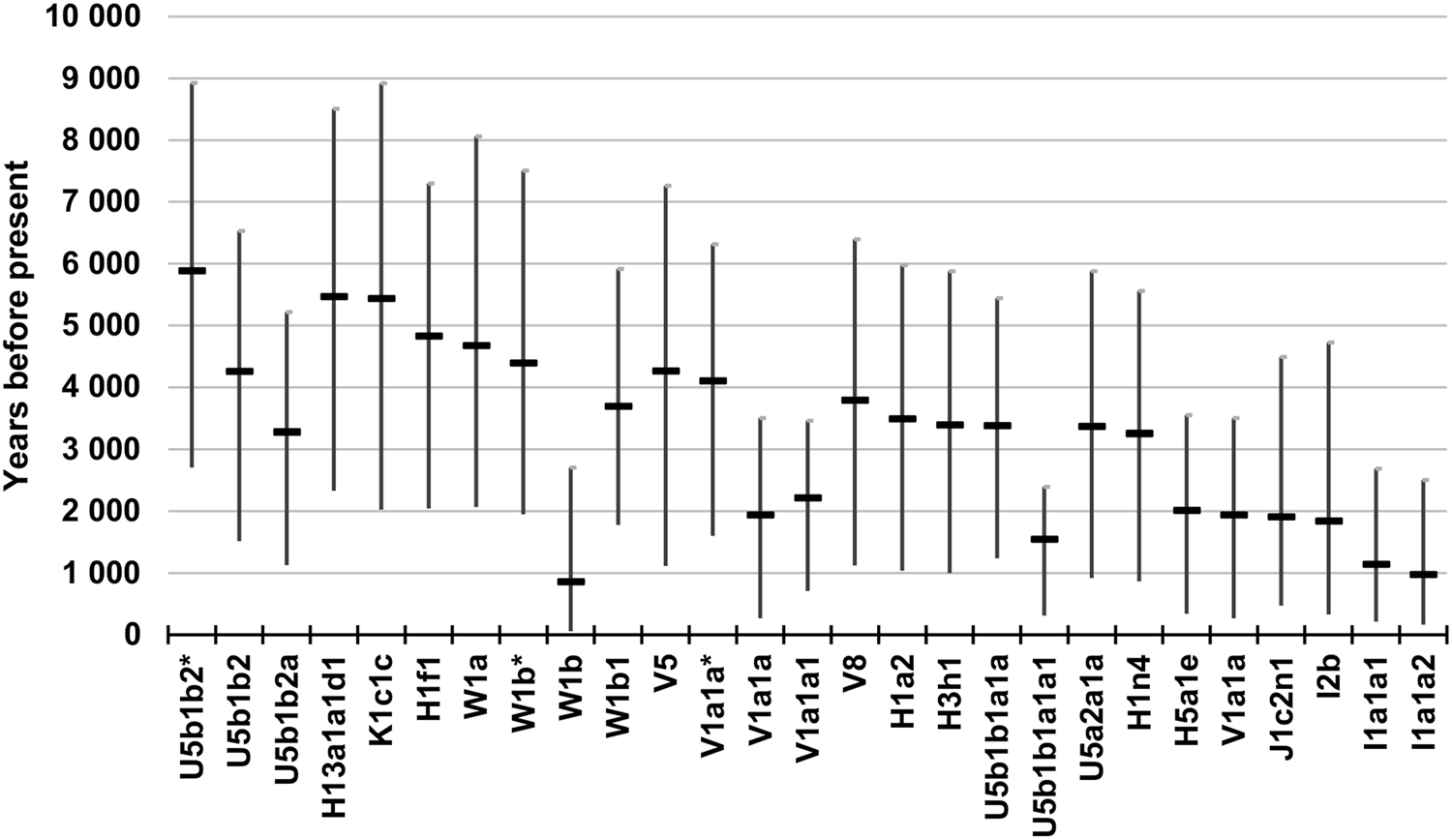
Median age estimates with 95% highest posterior density for Finn-characteristic subhaplogroups.

Ages for the Finn-characteristic haplogroups, estimated using the mutation rates above, are presented in Fig. 1 and Supplementary Table S8. The oldest subhaplogroup, U5b1b2*, dates back almost 6,000 years (median 5,883 years with 95% highest posterior density [2,708; 8,923] years), while the youngest subhaplogroup, W1b, is estimated to be less than 900 years old (median 858 years with 95% highest posterior density [57; 2,710] years). For a notable proportion of subhaplogroups (i.e. haplogroups H1n4, U5b1b2a, U5a2a1a, U5b1b1a1a, H3h1, H1a2, W1b1, V8, V1a1a*, U5b1b2, V5, W1b*, W1a, H1f1, K1c1c and H13a1a1d1) the median age estimate ranged from 3,300–5,500 ybp.

### Past population sizes: Bayesian skyline plots

**Figure 2:**
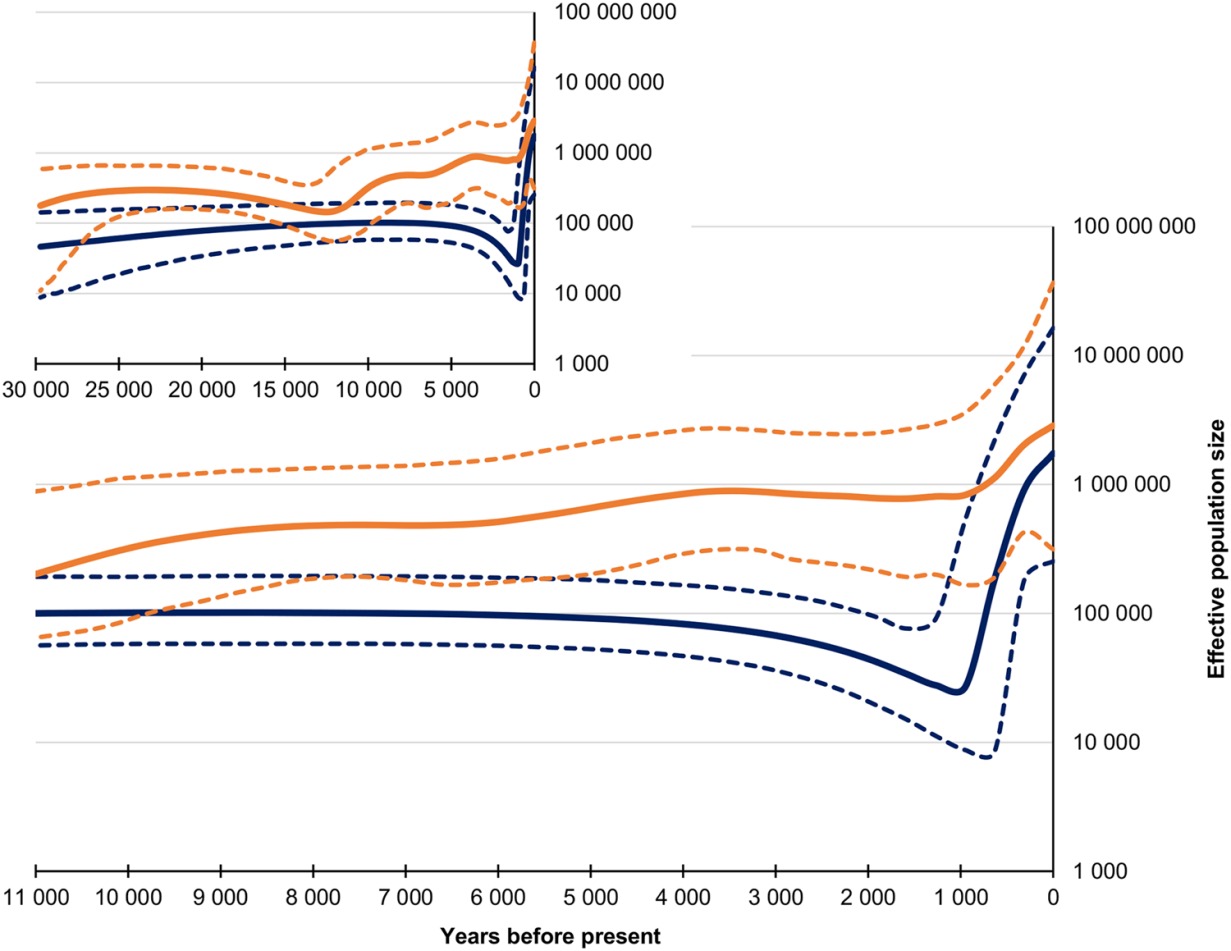
Effective population size (*N*_*e*_) comparison for Finn-characteristic (dark blue, N = 281) and non-Finn-characteristic (orange, N = 562) sequences. The larger graph zooms in on the last 11,000 years. In both graphs on the X-axis is time as years before present and on the Y-axis is effective population size represented on a logarithmic scale. The continuous center lines represent the mean for the *N*_*e*_, and the dotted lines are the 95% highest posterior density intervals.

In order to analyze population size changes over time, BSPs were constructed for Finn-characteristic and for other haplogroups (Fig. 2). For the non-Finn-characteristic haplogroups the increase in effective population size (*N*_*e*_) was relatively stable until around 4,000 years before present when the increase stops. The *N*_*e*_ was constant between 4,000–1,000 years before present after which it started to increase rapidly. Instead, for the Finn-characteristic haplogroups, the *N*_*e*_ was relatively stable until it started to decrease around 5,000–4,000 years before present. The lowest point, below one third of the previous stable *N*_*e*_, was reached around 1,000 ybp. The growth for Finn-characteristic haplogroups during the last 1,000 years was exponential, and the present *N*_*e*_ is over sixty times larger compared to the *N*_*e*_ of the lowest point. The present *N*_*e*_ for Finn-characteristic haplogroups is two thirds of the *N*_*e*_ for other haplogroups.

## Discussion

This study shows that the mitochondrial genome pool can harbor signals that are not discernible in lower resolution data sets. In fact, the patterns would have not been revealed without dissection of the complete genome data into local (“Finn-characteristic”) and non-local components. The contemporary complete mtDNA genome data set showed diversity values, haplogroup distribution, and (deduced) past population sizes similar to those of many other European populations. However, segregating the sequences based on their inclusion in “local” and “non-local” haplogroups revealed diversity patterns in our model population of Finland. This approach provided an opportunity for closer assessment of the maternal past and yielded results that show closer overall resemblance to those obtained with other markers such as the Y-chromosome.

Interestingly, a large number of the haplogroups in the data is actually rare elsewhere in Europe. It is notable that these Finn-characteristic haplogroups, in which >75% of the mtDNAs were observed only in Finland, are at different depths in the global mtDNA phylogeny (ranging from e.g. V8 to H13a1a1d1). These lineages may have i) arisen in Finland and drifted to considerable frequency locally (autochtonous lineages); ii) arisen somewhere else but survived only in Finland by chance; and/or iii) these lineages are present in other populations, but have not been sampled. There is insufficient evidence to indicate which of these possibilities (or combinations thereof) best explain the haplogroups, and hence the term “Finn-characteristic” was applied instead of the term “autochtonous” that would imply endemic history in Finland. However, the latter alternative (non-representative sampling) was considered unlikely given that the analysis was performed on the (sub)haplogroup level and that the number of complete mtDNA sequences recorded in GenBank is rather high (~30,000). When considering this bias it is worth noting that, as estimated from the Human Mitochondrial DataBase, Europeans are overrepresented in the GenBank (Supplementary Table S1), which should render the latter possibility less likely.

Another conspicuous difference is seen in the estimated past population sizes among the total data, Finnish, and non-Finnish haplogroups. The total data show development which is in concordance with previously published patterns for many European populations (including Finland^50^), or the Neolithic-associated H-haplogroup^51^. The deduced population sizes started to increase strongly in early Holocene c. 10,000–8,000 ybp, with an even sharper rise during the last few centuries. This increase has been associated with the Neolithic revolution and surplus nutrition produced after adopting agriculture. A general notion is that the hunter-gatherer populations show signatures of constant population sizes, different from the exponential growth patterns in the Neolithic cultures. A “Neolithic” pattern also was observed using the (Finnish) sequences in non-Finn-characteristic haplogroups. The Finn-characteristic haplogroups, however, showed a starkly different pattern, with rapidly decreasing population sizes throughout most of the Holocene, until a period of steep growth that started only as late as around 1,000 ybp. This difference between ‘local’ and ‘immigrant’ BSP curves is statistically significant. The results also suggest that a substantial reduction of population size dating took place around 2,000–1,000 years before present. This decrease in the estimated effective population sizes also was observed with the more stringent 90% cut-off limit used to delineate the Finnish haplogroups (Supplementary Fig. S5).

The population size reduction appears clear in the BSPs based on the Finn-characteristic haplotypes. Nevertheless, the possibility of this signal being an artefact must be considered. The BSP method assumes panmictic population, and violations, especially population structure, may produce similar signals as those of reductions in population size^52–54^. The magnitude of this problem obviously depends on the level of divergence between the (ignored) subpopulations, which in case of modern human mtDNA data usually is relatively small in Europe. However, a number of studies have demonstrated genetic substructure in Finland (see Introduction). In order to explore the possibility of spurious population change signals caused by a structure effect, the BSPs were constructed for modern HVR1 + 2 data sampled in the identified “subpopulations” in Finland^9^. The results (Supplementary Fig. S6) do not indicate a notable structure effect. The BSPs for the two subpopulations and the pooled population are similar, and none of them show population decline of the same magnitude that is observed in the Finn-characteristic haplotypes. This observation would tend to support that the overall declining pattern is real.

Two additional observations in the obtained graphs should be noted. First, the dip in the BSPs of Finn-characteristic haplotypes before the final population expansion must be treated with some caution, as similar patterns have been reported, e.g., from the Pacific herring (see Discussion in ref. 55). Secondly, it is worth noting that the values of estimated effective population may not be “real”, but can be treated as trends. The effective population size can be defined in a number of ways, and the issue is complex^56^.

Assuming the obtained results reflect the past population history, it is likely that both the high proportion of Finn-characteristic haplogroups and the reduced past population sizes arise from a small number of founders and relative genetic isolation for a rather long period of time. High levels of drift due to small population sizes and isolation-by-distance within Finland may have contributed strongly to the genetic diversity. While population bottlenecks have been suggested as a causal factor for Y-chromosomal and autosomal diversity patterns in Finland, and observations also in other types of data^57^, this pattern has rarely been deduced from mitochondrial DNA data. An exception is the study by Sajantila *et al*. 1996^6^, in which a bottleneck, dated to around 3,900 ybp, was inferred from the mitochondrial control region data. Sajantila *et al*. suggested that this bottleneck has influenced mtDNA diversity, but that mutations at rapidly evolving sites have generated diversity to levels observed in other parts of Europe. It is notable that, as in this study herein, the bottleneck was only visible after restricting the data into a subset of the total based on the variability of sites. A similar approach has been successfully employed by Fagundes *et al*. 2008^2^.

Although the approaches by Sajantila *et al*. 1996^6^ and this study are in principle similar in observing patterns only in a restricted data set, they do convey different explanations. Considering the evolutionary forces, Sajantila *et al*. (1996) offer mutation as an explanation for the discrepancy between the high mitochondrial and lower Y-chromosomal diversity in Finland. However, there are several factors that may limit the role of mutation. First, the differences between Y-chromosomal and mtDNA mutation rates are not as large as the diversity differences between the two markers. Second, the use of pedigree rates has been shown to result in overestimates when considering data in a population sample^58^. Third, observing lower diversity in slowly evolving sites is likely to result from restricted haplogroup distribution, which is not a particularly conspicuous feature of the Finnish mtDNA pool. Alternatively, the current results, showing lower diversity (and hence population sizes) in the Finn-characteristic haplogroups, would suggest migration, instead of mutation, as an important source of relatively high mtDNA diversity in modern Finns. This notion would be in line with some simulation studies emphasizing constant, low-level gene flow in Finland^59, 60^.

The estimated haplogroup ages may shed light on the past events in Finland. Diversity within the non-Finnish haplogroups was shaped largely by distant environmental conditions and human population histories and can tell little about the Finns. Instead, the ages of the Finn-characteristic haplogroups range from 858 [57; 2,710] years for haplogroup W1b to 5,883 [2,708; 8,923] years for haplogroup U5b1b2*. In general, there appears to be two loose and largely overlapping clusters among the Finn-characteristic haplogroups: the first between 1,000–2,000 ybp and the second around 3,300–5,500 ybp. The age of the older cluster coincides temporally with the arrival of the Corded-Ware culture and, notably, the spread of agriculture in Finland. The arrival and spread of agriculture, temporally corresponding with the age estimates for most of the haplogroups characteristic of Finns, might be a sign of population size increase enabled by the new mode of subsistence, resulting in reduced drift and accumulation of genetic diversity in the population. Despite the documented demic diffusion associated with the Neolithic transition (see refs 61 and 62), it is likely that cultural diffusion also has taken place (see ref. 63). This pattern would explain the observed ‘Neolithic’ increase of diversity in both hunter-gatherer (U and V) and farmer (H, J and K) associated Finn-characteristic haplogroups. It should be noted that our data set might be slightly biased towards northern Finland, where the Corded-Ware culture did not reach. Note that the (Mesolithic/Neolithic) classification of haplogroup V is contentious, but here it is included in the hunter-gatherer pool due to its high frequency among the Saami, the indigenous reindeer herders in northern Finland. While the mtDNA diversity of the Saami is not the focus of the study herein, it is important to note that their history in Finland predates that of Finns^64^ and that there has been (mtDNA) admixture between the Saami and the Finns (Evidence for mtDNA admixture between the Finns and the Saami in Meinilä *et al*.)^65^.

Another insight in the past population sizes in Finland is based on radiocarbon-dated archaeological findings in different time periods. These analyses suggest two prehistoric population peaks in Finland, the Stone Age peak (c. 5,500 ybp) and the Metal Age peak (~1,500 ybp)^57, 66, 67^. Both of these peaks were followed by a population decline, which appears to have reached its ebb around 3,500 ybp. These developments are not distinguishable in the BSPs. However, these ages correspond well to the two haplogroup age clusters described above. The presumably less severe Iron Age population bottleneck seen in the archaeological data, 1,500–1,300 ybp^57, 67^, temporally coincides with the population size reduction visible for the Finn-characteristic subhaplogroups.

## Conclusion

The palimpsest nature of today’s genetic diversity may hide retrievable signals of past demography, only visible in subsets of data. In the case of Finland, dissection of the total variation into local and non-local variation components, enable visualization of demographic history patterns that are compatible with results deduced based on diverse data. These results also help to understand the apparent diversity differences between mitochondrial, Y-chromosomal and autosomal markers described in the Finnish population. Such methods that allow the deduction of the past from modern genetic data are especially helpful in regions where ancient DNA studies are limited due to paucity of archaeological bone material.

## Electronic supplementary material

Supplementary information

Supplementary dataset
