# Supplementary material for: Identification and analysis of mtDNA genomes attributed to Finns reveal long-stagnant demographic trends obscured in the total diversity
Source: Sci Rep. 2017 Jul 21;7:6193. doi: 10.1038/s41598-017-05673-7 (PMC5522469; doi:10.1038/s41598-017-05673-7)
Supplement: Supplementary file 1 — Supplementary information [file 41598_2017_5673_MOESM1_ESM.pdf]

Identification and analysis of mtDNA genomes attributed to Finns reveal long-stagnant demographic trends obscured in the total diversity

Scientific Reports

Översti S*, Onkamo P, Stoljarova M, Budowle B, Sajantila A, Palo JU

***Corresponding author, University of Helsinki, sanni.oversti@helsinki.fi**

Supplementary Information

Supplementary Table S1. The origins by continent of complete mtDNA genomes in GenBank estimated from the Human Mitochondrial DataBase, HmtDB (http://www.hmtdb.uniba.it/hmdb/onlinehelp/stats.html)

Continent	Complete mitochondrial genomes in HmtDB	Percentage of all sequences deposited in HmtDB (N_{total}=29,861)
Africa	3,014	10.1
America	2,255	7.6
Asia	7,440	24.9
Europe	8,282	27.7
Oceania	1,523	5.1
Undefined continent	7,347	24.6
All continents	29,861	100.0

Supplementary Table S2. Complete mtDNA genomes in GenBank from nearby populations based on Human Mitochondrial DataBase (HmtDB) searches

Population	Number of sequences
Belarus	45
Czech	60
Denmark	2,888
Estonia	10 + 114*
France	200
Germany	366
Hungary	23
Latvia	3
Lithuania	27
Norway	74
Poland	163
Russia	1,219
Slovakia	51
Slovenia	9
Sweden	111
Saami	21
Ukraine	43

*[1]

Supplementary Table S3. Ancient complete mtDNA genomes used in this study as calibration points for dating.

GenBank ID	Haplogroup	cal ^{14}C ybp	Origin	Reference
KC521455	U5b1a	8,054 ± 127	Loschbour	[2]
KC521457	U5b1	14,020 ± 150	Oberkassel	[2]
KC521458	U	31,155 ± 85	Dolci Vestonice	[2]
KC521459	U8	31,155 ± 85	Dolci Vestonice	[2]
KC521454	B4c1a	8,180 ± 140	Boshan	[2]
KF523402	H5	3,666 ± 20	Blätterhöhle (Neolithic)	[3]
KF523403	H1c3	3,418 ± 63	Blätterhöhle (Neolithic)	[3]
KF523404	U5b2b	3,922 ± 60	Blätterhöhle (Neolithic)	[3]
KF523405	H5	3,513 ± 102	Blätterhöhle (Neolithic)	[3]
KF523406	U5b2b2	3,603 ± 49	Blätterhöhle (Neolithic)	[3]
KF523407	U5a2c3	8,652 ± 58	Blätterhöhle (Mesolithic)	[3]
KC521456	T2b1	690 ± 39	”Cro-Magnon 1” France (Medieval)	[2]

Supplementary Table S4. Haplogroup frequencies in Finland based on this study. In total 240 subhaplogroups were observed. Finn-characteristic haplogroups indicated with blue color.

Haplogroup	Number of sequences	Frequency (%)
**D***	3	0.4
D5a3a1a	3	0.4
**G***	1	0.1
G3a1	1	0.1
**H***	311	36.9
H	5	0.6
**H1***	120	14.2
H1	17	2.0
H1a	7	0.8
H1aa1	1	0.1
H1ag1	1	0.1
H1am	1	0.1
H1ap1	2	0.2
H1a2	21	2.5
H1a8	3	0.4
H1a8a	1	0.1
H1b	1	0.1
H1b1	3	0.4
H1b1d	2	0.2

H1b1i	4	0.5
H1b5	1	0.1
H1c	2	0.2
H1c1	1	0.1
H1c22	1	0.1
H1c3b	2	0.2
H1c9	3	0.4
H1f1	27	3.2
H1f1a	3	0.4
H1h1	2	0.2
H1q	1	0.1
H1q1	3	0.4
H1n4	10	1.2
**H2***	31	3.7
H2a	1	0.1
H2a1	12	1.4
H2a1c	3	0.4
H2a1f2	2	0.2
H2a1m	2	0.2
H2a1n	1	0.1
H2a2a1b	3	0.4
H2a2b3	4	0.5
H2a3a	1	0.1
H2a3a1	2	0.2
**H3***	27	3.2
H3	3	0.4
H3as	1	0.1
H3b	2	0.2
H3b6a	3	0.4
H3h1	14	1.7
H3h3	1	0.1
H3h5	2	0.2
H3t	1	0.1
**H4***	11	1.3
H4a1a1	3	0.4
H4a1a1a	8	0.9
**H5***	10	1.2
H5a1a	2	0.2
H5a1e	4	0.5
H5a8	2	0.2
H5c1a	2	0.2
**H6***	8	0.9
H6a1a	3	0.4
H6a1a3	3	0.4
H6a1a4	1	0.1
H6a1a8a	1	0.1

**H7***	6	0.7
H7	1	0.1
H7a	1	0.1
H7a1a	1	0.1
H7a1b	1	0.1
H7b	1	0.1
H7c3	1	0.1
**H10***	6	0.7
H10e	4	0.5
H10e1a	1	0.1
H10f	1	0.1
**H11***	12	1.4
H11	1	0.1
H11a	3	0.4
H11a1	4	0.5
H11a2	3	0.4
H11a2a2	1	0.1
**H13***	20	2.4
H13a1a1d	3	0.4
H13a1a1d1	11	1.3
H13a1d	1	0.1
H13a2	1	0.1
H13a2b	2	0.2
H13a2b5	2	0.2
H14*	1	0.1
H14a2c	1	0.1
**H15***	2	0.2
H15	2	0.2
**H17***	1	0.1
H17a	1	0.1
**H24***	5	0.6
H24a	5	0.6
**H26***	1	0.1
H26c	1	0.1
**H27***	9	1.1
H27a	8	0.9
H27e	1	0.1
**H28***	7	0.8
H28a	5	0.6
H28a2	2	0.2
**H31***	3	0.4
H31	3	0.4
**H35***	3	0.4
H35	3	0.4
**H36***	1	0.1
H36	1	0.1

**H39***	7	0.8
H39	2	0.2
H39c	5	0.6
**H45***	5	0.6
H45	1	0.1
H45a	4	0.5
**H49***	2	0.2
H49	1	0.1
H49a	1	0.1
**H85***	2	0.2
H85	2	0.2
**H95***	1	0.1
H95a	1	0.1
**H104***	5	0.6
H104a	5	0.6
**HV***	4	0.5
HV0a1	1	0.1
HV15	2	0.2
HV9	1	0.1
**I***	24	2.8
**I1***	17	2.0
I1a1a	8	0.9
I1a1a1	5	0.6
I1a1a2	4	0.5
**I2***	6	0.7
I2a1a	2	0.2
I2b	4	0.5
**I5***	1	0.1
I5a1	1	0.1
**J***	54	6.4
**J1***	41	4.9
J1b1a1	1	0.1
J1b1a1b	2	0.2
J1c1a	3	0.4
J1c1b2	1	0.1
J1c1d	1	0.1
J1c2	4	0.5
J1c2b5	2	0.2
J1c2c1	3	0.4
J1c2c2	1	0.1
J1c2n	5	0.6
J1c2n1	8	0.9
J1c3	2	0.2
J1c3k	1	0.1
J1c6	1	0.1
J1c7a	5	0.6

J1c8a1a	1	0.1
**J2***	13	1.5
J2a1a1a	2	0.2
J2a1a1a1	6	0.7
J2b1	2	0.2
J2b1a	1	0.1
J2b1a2	2	0.2
**K***	51	6.0
**K1***	47	5.6
K1a	1	0.1
K1a13a	2	0.2
K1a1a	1	0.1
K1a1b1b1	2	0.2
K1a1b2b	1	0.1
K1a2a2	2	0.2
K1a2c	1	0.1
K1a4a1a2a	1	0.1
K1a4a1a2b	3	0.4
K1a4a1c	1	0.1
K1a4a1f	1	0.1
K1b2a	1	0.1
K1b2a1	2	0.2
K1c1	8	0.9
K1c1c	18	2.1
K1c1f	1	0.1
K1c1h	1	0.1
**K2***	2	0.2
K2a4	2	0.2
K2b1a1a	2	0.2
**N***	1	0.1
N1a1a1a1	1	0.1
**R***	6	0.7
**R1***	2	0.2
R1a1	1	0.1
R1b1	1	0.1
**R2***	4	0.5
R2b	4	0.5
**T***	37	4.4
**T1***	12	1.4
T1a1	7	0.8
T1a1b	1	0.1
T1a1q	2	0.2
T1b1	2	0.2
**T2***	25	3.0
T2	1	0.1
T2a1	1	0.1

T2a1b	1	0.1
T2a1b1a1a2	1	0.1
T2b	4	0.5
T2b36	2	0.2
T2b3d	1	0.1
T2b4	4	0.5
T2c1c2	2	0.2
T2c1d	1	0.1
T2e1	1	0.1
T2f1a1	5	0.6
T2f2	1	0.1
**U***	190	22.5
**U1***	1	0.1
U1b2	1	0.1
**U2***	8	0.9
U2e1a1	3	0.4
U2e1a1a	3	0.4
U2e1b1	1	0.1
U2e2a1a	1	0.1
**U3***	1	0.1
U3a1	1	0.1
**U4***	10	1.2
U4a2	3	0.4
U4a2a	1	0.1
U4a2b	1	0.1
U4d1a1	2	0.2
U4d1a1a	3	0.4
**U5***	152	18.0
U5a1	1	0.1
U5a1a1	2	0.2
U5a1a1b	2	0.2
U5a1a1e	1	0.1
U5a1a1h	2	0.2
U5a1b	1	0.1
U5a1b1	1	0.1
U5a1b1c1	2	0.2
U5a1b1h	8	0.9
U5a1b3a1	9	1.1
U5a2a1a	13	1.5
U5a2a1e	5	0.6
U5a2a2a	2	0.2
U5a2b	1	0.1
U5a2b3a	1	0.1
U5a2d1	1	0.1
U5a2d1a	1	0.1
U5b1b1	4	0.5

U5b1b1a	24	2.8
U5b1b1a1	3	0.4
U5b1b1a1a	9	1.1
U5b1b1a1a1	7	0.8
U5b1b1a1b	2	0.2
U5b1b1a3	1	0.1
U5b1b2	23	2.7
U5b1b2a	12	1.4
U5b1b2b	2	0.2
U5b1e1	4	0.5
U5b2a	1	0.1
U5b2a1a	1	0.1
U5b2a1a1b	3	0.4
U5b2a1a1d	1	0.1
U5b2a5a	2	0.2
**U7***	6	0.7
U7b2	6	0.7
**U8***	12	1.4
U8a1a	1	0.1
U8a1a1	5	0.6
U8a1a1b1	6	0.7
**V***	73	8.7
V	6	0.7
**V1***	29	3.4
V1a	1	0.1
V1a1	10	1.2
V1a1a	6	0.7
V1a1a1	12	1.4
**V2***	2	0.2
V2a1a	2	0.2
**V5***	9	1.1
V5	9	1.1
**V7***	16	1.9
V7a1	16	1.9
**V8***	11	1.3
V8	11	1.3
**W***	65	7.7
W	1	0.1
**W1***	59	7.0
W1	2	0.2
W1a	35	4.2
W1b	4	0.5
W1b1	14	1.7
W1e1	4	0.5
**W4***	3	0.4
W4a1	3	0.4

**W6***	2	0.2
W6	1	0.1
W6a	1	0.1
**X***	12	1.4
**X2***	12	1.4
X2b11	1	0.1
X2b4a1	1	0.1
X2b9	1	0.1
X2c1	6	0.7
X2c1a	2	0.2
X2c1e	1	0.1
**Z***	11	1.3
**Z1***	11	1.3
Z1a1a	11	1.3

Supplementary Table S5. Main haplogroup frequencies estimated from HVR1+2 data presented in Palo et al. 2009[4] and this study.

**Haplogroup**	**N HVR1+2 (N_{total}=832)**	**% HVR1+2**	**N This study (N_{total}=843)**	**% This study**
H	276	33.2	311	36.9
>H1	94	11.3	120	14.2
HV	5	0.6	4	0.5
I	10	1.2	24	2.8
J	46	5.5	54	6.4
K	46	5.5	51	6.0
N	0	0.0	1	0.1
R	6	0.7	6	0.7
T	51	6.1	37	4.4
U	202	24.3	190	22.5
>U5	175	21.0	152	18.0
>>U5a	53	6.4	53	6.3
>>U5b	122	14.7	99	11.7
V	30	3.6	73	8.7
>V1	9	1.1	29	3.4
>V7	14	1.7	16	1.9
W	31	3.7	65	7.7
X	15	1.8	12	1.4
Z	4	0.5	11	1.3
Others/undefined	110	13.2	0	0.0

Supplementary Table S6. Proportion of main haplogroups and Finn-characteristic haplogroups within each dataset (i.e. 1000 Genomes[5], Behar et al. 2012[6], Finnilä et al. 2001[7], Raule et al. 2014[8], Soini et al. 2012[9] and Soini et al. 2013[10]) from Finland.

**Haplogroup**	**% 1000 Genomes (N_{total}=93)**	**% Behar et al. 2012 (N_{total}=94)**	**% Finnilä et al. 2001 (N_{total}=192)**	**% Raule et al. 2014 (N_{total}=293)**	**% Soini et al. 2012 ((N_{total}=64)**	**% Soini et al. 2013 (N_{total}=79)**
D	0.0	1.1	0.0	0.7	0.0	0.0
G	0.0	0.0	0.0	0.3	0.0	0.0
H	36.6	53.2	16.1	45.1	35.9	40.5
HV	1.1	0.0	0.0	1.0	0.0	0.0
I	2.2	1.1	6.8	1.0	4.7	2.5
J	8.6	4.3	8.9	6.1	3.1	5.1
K	6.5	7.4	6.3	5.8	4.7	3.8
N	0.0	0.0	0.0	0.3	0.0	0.0
R	0.0	1.1	0.0	1.4	1.6	0.0
T	3.2	3.2	5.7	3.8	7.8	3.8
U	35.5	21.3	16.1	20.1	23.4	26.6
V	4.3	3.2	14.1	7.5	15.6	8.9
W	1.1	2.1	19.3	5.1	3.1	6.3
X	1.1	0.0	2.1	1.7	0.0	2.5
Z	0.0	2.1	4.7	0.0	0.0	0.0
Finn-characteristic	39.8	20.2	45.3	24.9	37.5	32.9

Supplementary Table S7. Base frequencies, transition/transversion rates, value for gamma distribution shape parameter *α* and proportion of invariant sites for each of the four schemes estimated based on phylogenetic analyses.

**Scheme number**	**Substitution model**	**Parameter**	**Mean ± stdev**
**1**	TrN + Γ + Inv	*κ1*	20.43 ± 6.78
Nucleotides 1–576 and 16024–16569		*κ2*	23.41 ± 7.10
		Frequency A	0.29 ± 0.01
		Frequency C	0.34 ± 0.01
		Frequency G	0.14 ± 0.01
		Frequency T	0.23 ± 0.01
		α	0.62 ± 0.29
		pInv	0.82 ± 0.04
**2**	HKY+ Γ + Inv	*κ*	27.14 ± 15.24
rRNA, tRNA		Frequency A	0.35 ± 0.01
		Frequency C	0.25 ± 0.01
		Frequency G	0.17 ± 0.01
		Frequency T	0.23 ± 0.01
		α	0.45 ± 0.42
		pInv	0.94 ± 0.03
**3**	TrN + Γ + Inv	*κ1*	58.32 ± 22.47
PC1, PC2		*κ2*	15.00 ± 5.99
		Frequency A	0.27 ± 0.01
		Frequency C	0.30 ± 0.01
		Frequency G	0.14 ± 0.01
		Frequency T	0.29 ± 0.01
		α	0.42 ± 0.43
		pInv	0.87 ± 0.05
**4**	TrN + Γ + Inv	*κ1*	80.72 ± 40.34
PC3		*κ2*	42.56 ± 21.08
		Frequency A	0.35 ± 0.01
		Frequency C	0.39 ± 0.01
		Frequency G	0.07 ± 0.01
		Frequency T	0.18 ± 0.01
		α	0.45 ± 0.44
		pInv	0.69± 0.14

κ = transition/transversion rate in HKY substitution model
κ1 = purine transition/transversion rate in TrN substitution model
κ2 = pyrimidine transition/transversion rate in TrN substitution model
α = shape parameter in gamma distribution
pInv = proportion of invariant sites

Supplementary Table S8. Median age estimate and 95% highest posterior density for Finn-characteristic subhaplogroups. For the comparison, divergence estimates by Behar et al. 2012 [6] are also presented.

**Haplogroup**	**Median (ybp)**	**95% Lower HPD (ybp)**	**95% Upper HPD (ybp)**	**Age estimate by Behar et al. 2012 (stdev) (ybp)**
H13a1a1d1	5,467	2,331	8,507	-
H1a2	3,488	1,039	5,974	2,908 (2,634)
H1f1	4,827	2,042	7,297	1,449 (2,216)
H1n4	3,252	861	5,560	4,174 (3,449)
H3h1	3,392	998	5,877	1,569 (2,081)
H5a1e	2,016	339	3,549	2,977 (2,737)
I1a1a1	1,142	215	2,691	-
I1a1a2	973	166	2,503	-
I2b	1,839	331	4,722	1,266 (4,538)
J1c2n1	1,910	472	4,490	-
K1c1c	5,433	2,026	8,919	1,508 (1,982)
U5a2a1a	3,365	917	5,876	1,235 (1,923)
U5b1b1a1a	3,379	1,236	5,439	1,356 (1,644)
U5b1b1a1a1	1,544	310	2,395	-
U5b1b2*	5,883	2,708	8,923	1,981 (2,782)
U5b1b2	4,256	1,517	6,532	-
U5b1b2a	3,279	1,128	5,217	-
V1a1a*	4,102	1,599	6,317	2,681 (3,764)
V1a1a	1,942	265	3,501	-
V1a1a1	2,214	713	3,457	-
V5	4,259	1,116	7,261	2,903 (4,139)
V8	3,787	1,122	6,395	2,903 (3,449)
W1a	4,676	2,070	8,057	3,011 (2,912)
W1b*	4,392	1,949	7,505	2,853 (2,282)
W1b	858	57	2,710	-
W1b1	3,688	1,782	5,913	1,635 (2,002)

Supplementary Figure S1. Neighbor-Joining tree for haplogroup H. Finnish sequences indicated with blue dot, others with red. Bootstrap values greater than 50 are shown. Estonian samples (EST15, EST45, EST75, EST27 and EST5) obtained from Stoljarova et al. 2016[1].

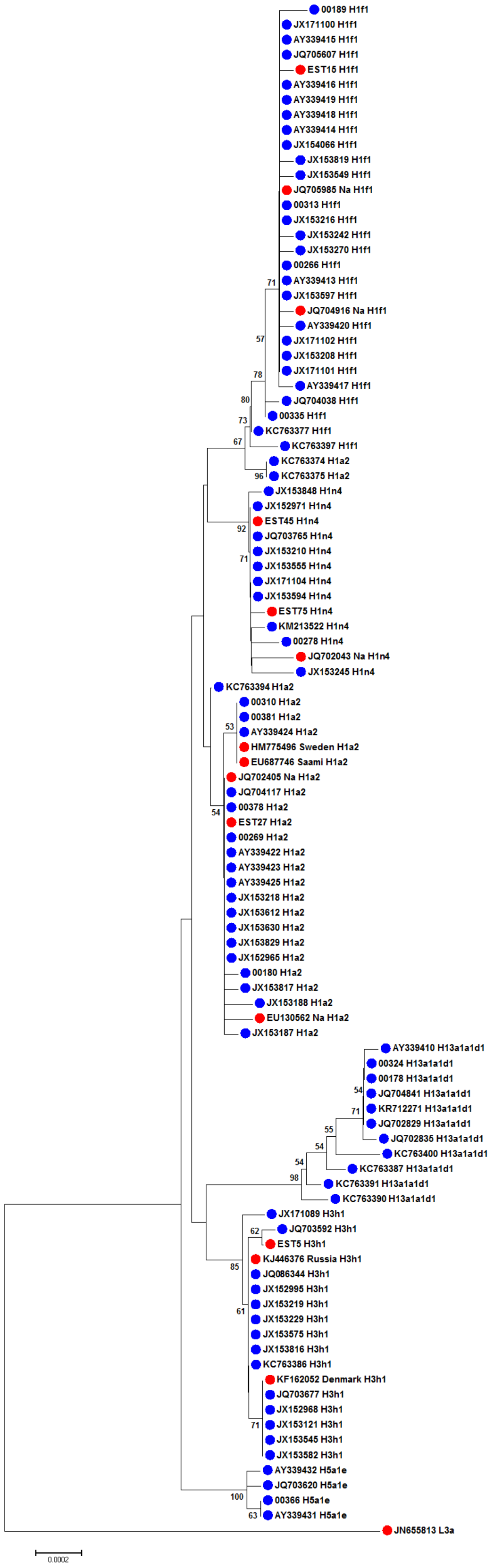

Supplementary Figure S2. Neighbor-Joining tree for haplogroup U. Finnish sequences indicated with blue dot, others with red. Bootstrap values greater than 50 are shown. Estonian samples (EST107, EST18, EST1) obtained from Stoljarova et al. 2016[1].

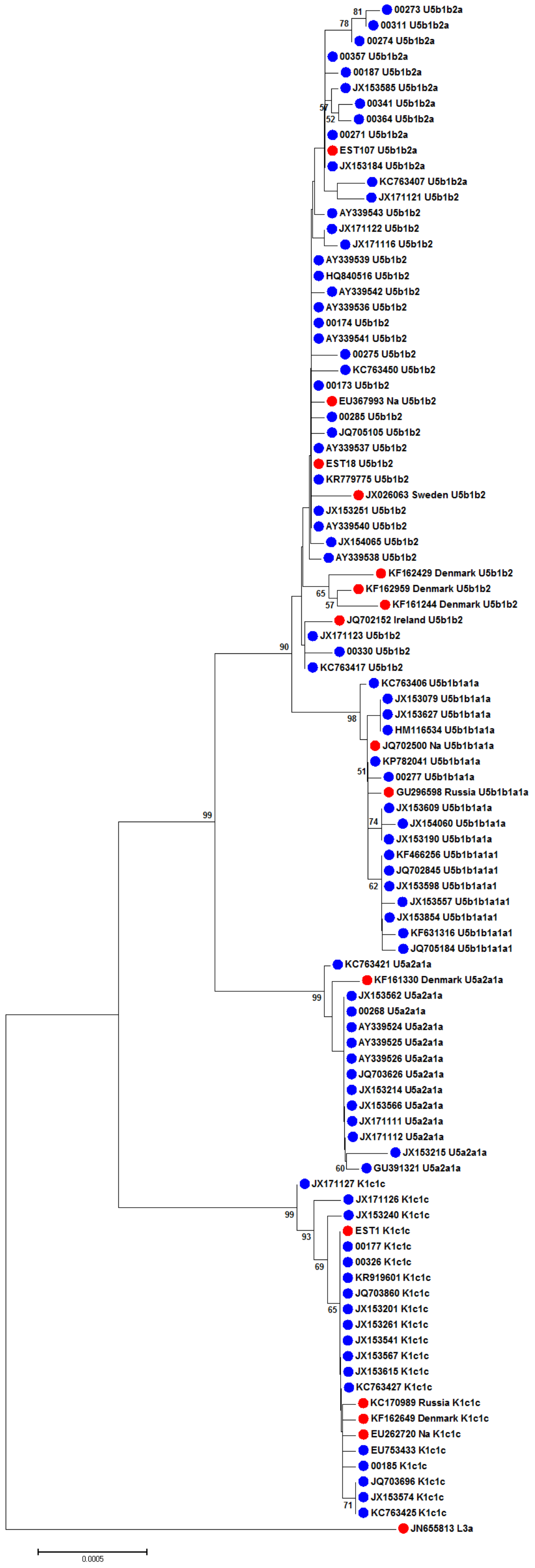

Supplementary Figure S3. Neighbor-Joining tree for haplogroup V. Finnish sequences indicated with blue dot, others with red. Bootstrap values greater than 50 are shown.

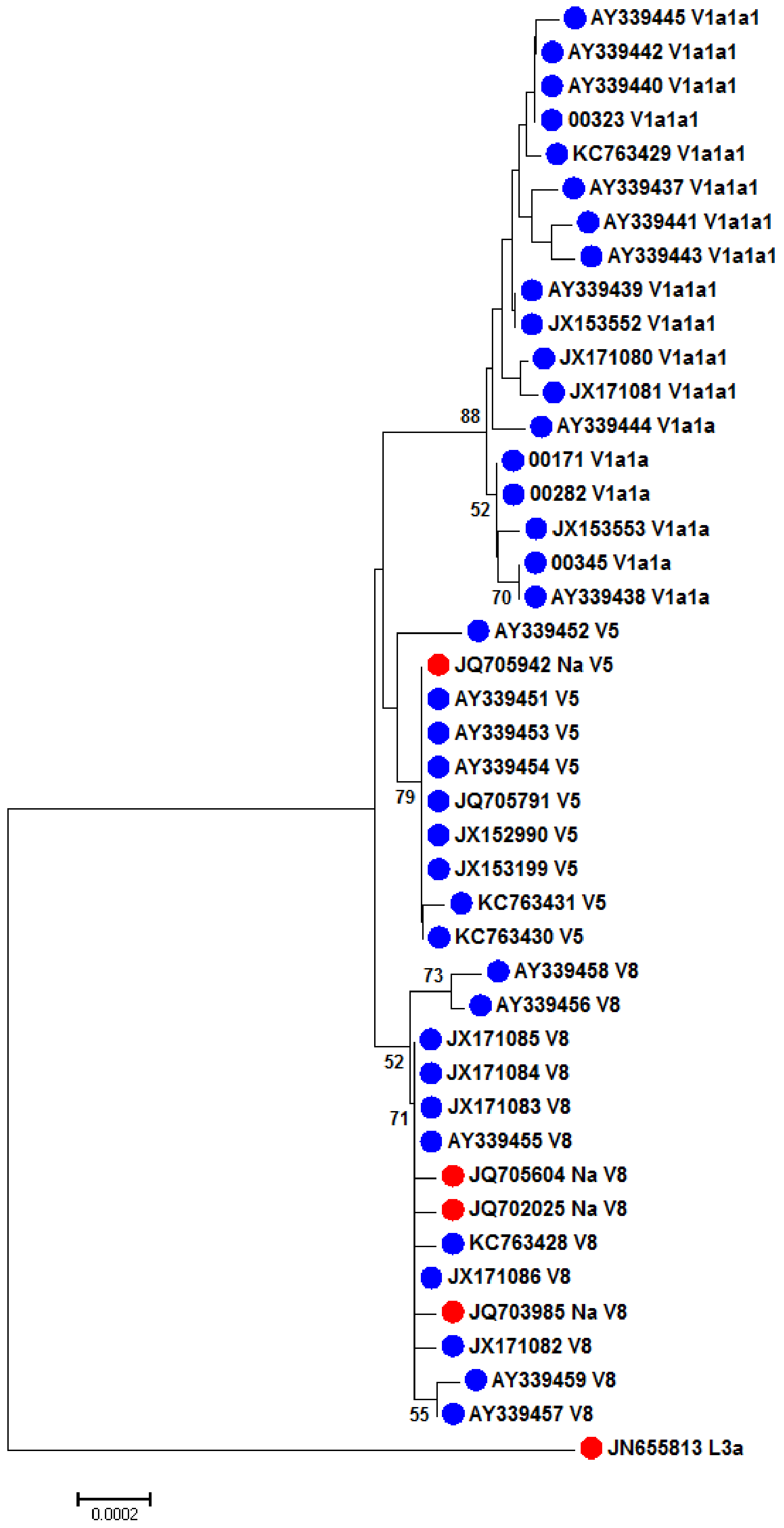

Supplementary Figure S4. Neighbor-Joining tree for haplogroup W. Finnish sequences indicated with blue dot, others with red. Bootstrap values greater than 50 are shown. Estonian sample (EST37) obtained from Stoljarova et al. 2016[1].

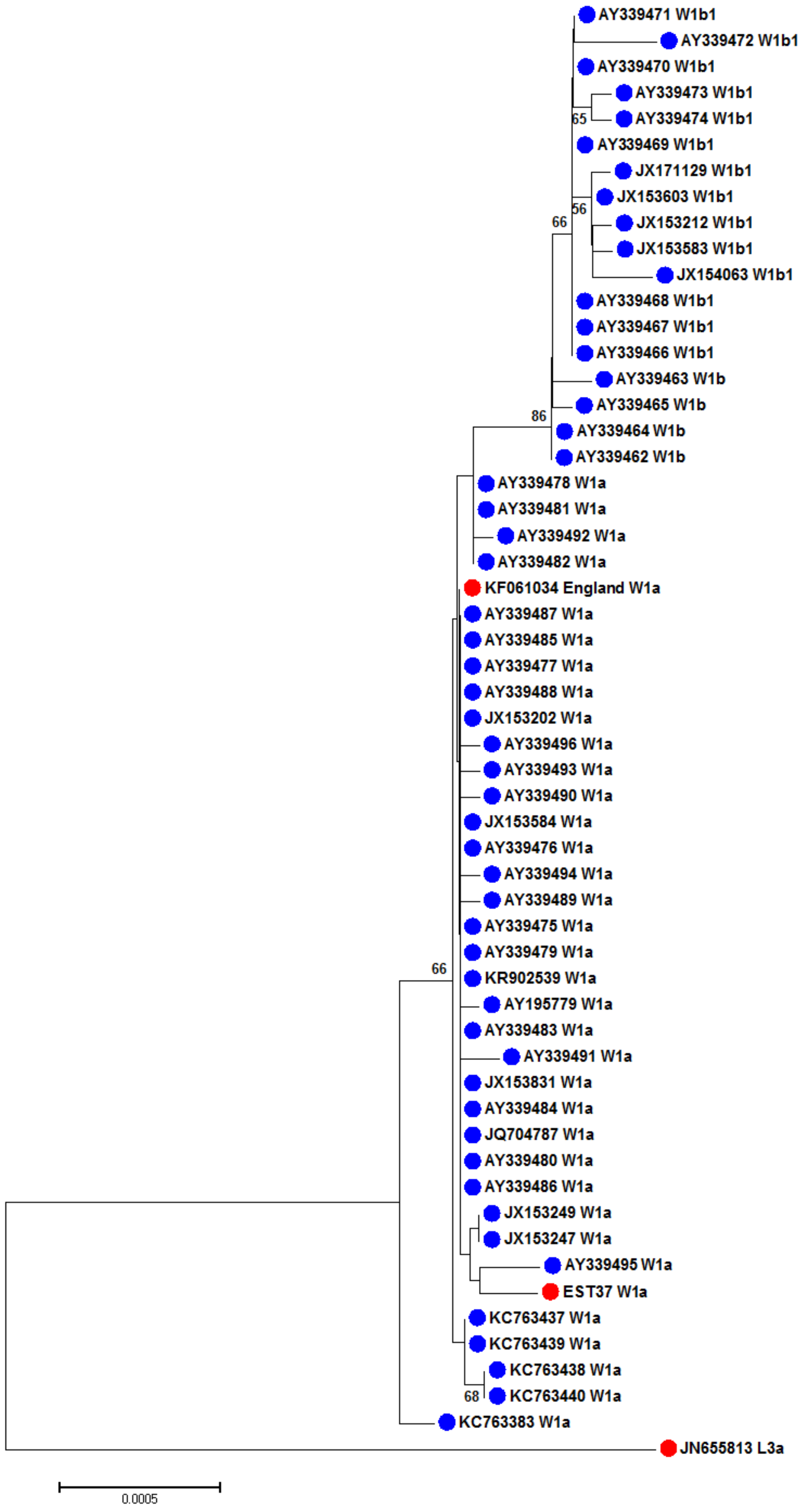

Supplementary Figure S5. Effective population size comparison for Finn-characteristic haplogroups by using the cut-off limit 75% (dark blue, N=281) and 90% (light blue, N=174). On the X-axis there is time as years before present and on the Y-axis there is effective population size represented on a logarithmic scale. The continuous center lines represent the mean for the N_e and the dotted lines are the 95% highest posterior density intervals.

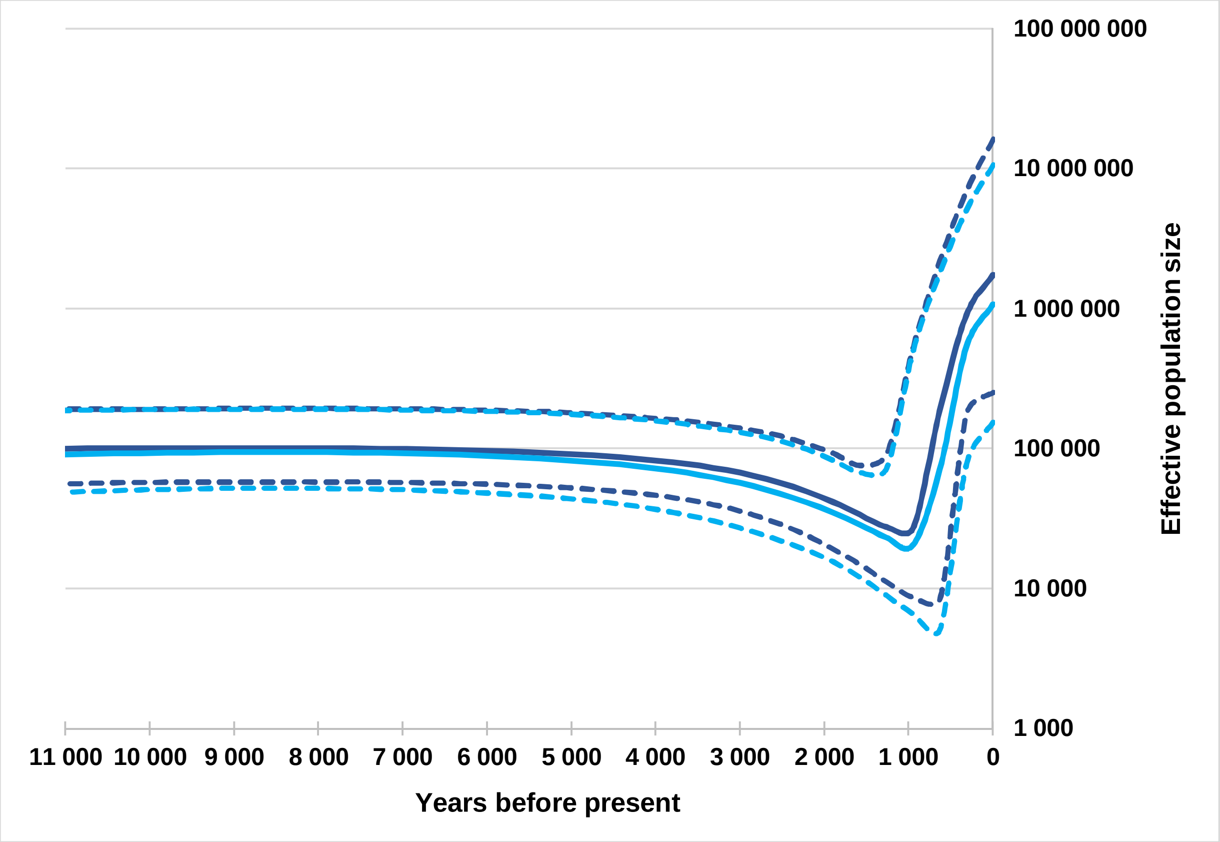

Supplementary Figure S6. Effective population size comparison for HVR1+2 data from Neuvonen et al. 2015[11]. BSPs were constructed separately for eastern Finland (yellow), southern Finland (green) and all data (grey) according to the Neuvonen et al. 2015[11]. On the X-axis there is time as years before present and on the Y-axis there is effective population size represented on a logarithmic scale. The continuous center lines represent the mean for the N_e and the dotted lines are the 95% highest posterior density intervals.

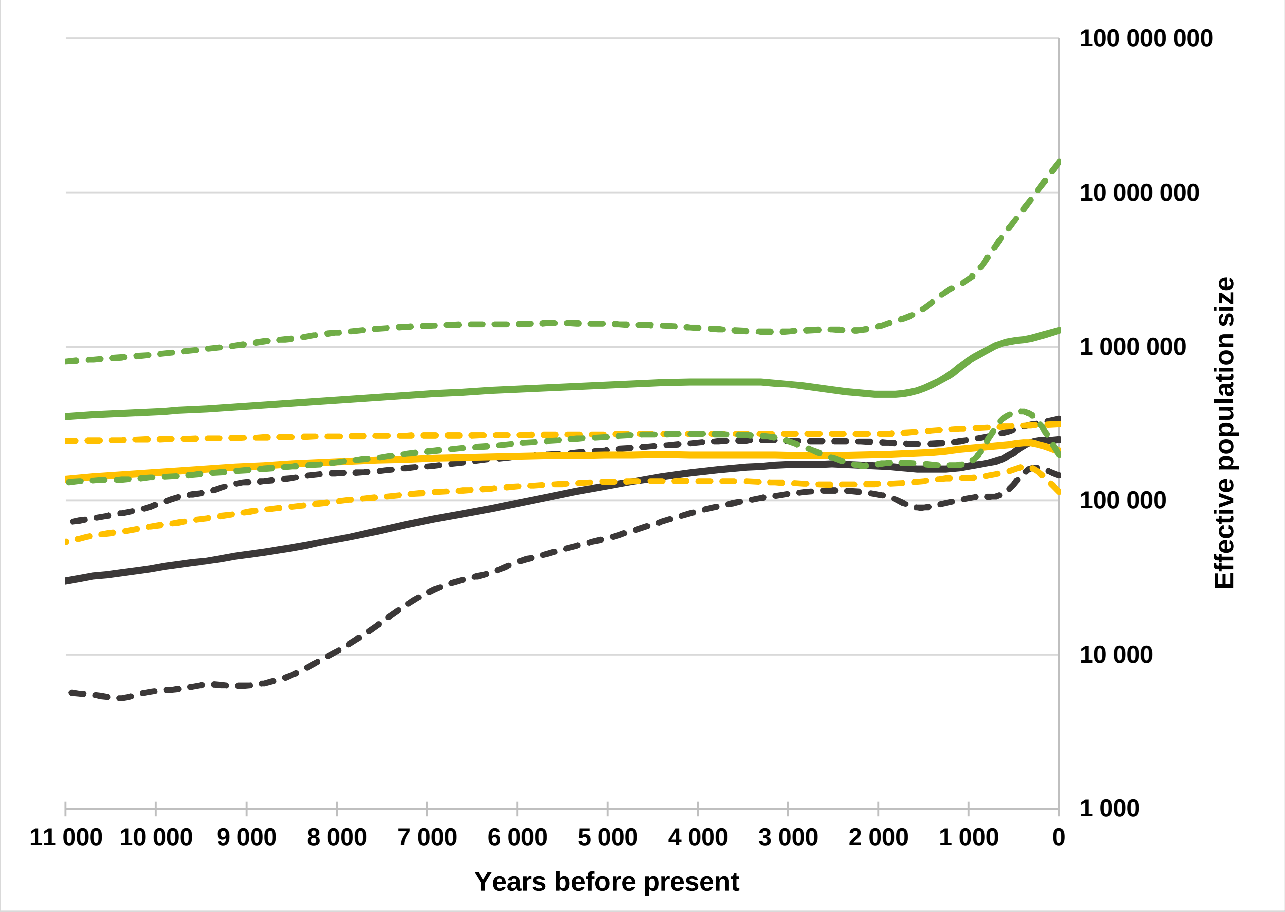

References

1. Stoljarova, M., King, J. L., Takahashi, M., Aaspõllu, A. & Budowle, B. Whole mitochondrial genome genetic diversity in an Estonian population sample. *Int. J. Legal Med.* **130**, 67-71 (2016).

2. Fu, Q. *et al*. A revised timescale for human evolution based on ancient mitochondrial genomes. *Current Biology* **23**, 553-559 (2013).

3. Bollongino, R. *et al*. 2000 years of parallel societies in Stone Age Central Europe. *Science* **342**, 479-481 (2013).

4. Palo J.U., Ulmanen I., Lukka M., Ellonen P. & Sajantila A. Genetic markers and population history: Finland revisited. *Eur. J. Hum. Genet.* **17**, 1336-1346 (2009).

5. 1000 Genomes Project Consortium. An integrated map of genetic variation from 1,092 human genomes. *Nature* **491**, 56-65 (2012).

6. Behar, D. M. *et al.* A “Copernican” reassessment of the human mitochondrial DNA tree from its root. *The American Journal of Human Genetics* **90**, 675-684 (2012).

7. Finnilä, S., Lehtonen, M. S. & Majamaa, K. Phylogenetic network for European mtDNA. *The American Journal of Human Genetics* **68**, 1475-1484 (2001).

8. Raule, N. *et al.* The co-occurrence of mtDNA mutations on different oxidative phosphorylation subunits, not detected by haplogroup analysis, affects human longevity and is population specific. *Aging cell* **13**, 401-407 (2014).

9. Soini, H. K., Moilanen, J. S., Finnila, S. & Majamaa, K. Mitochondrial DNA sequence variation in Finnish patients with matrilineal diabetes mellitus. *BMC Res. Notes* **5**, 350-0500-5-350 (2012).

10. Soini, H. K., Moilanen, J. S., Vilmi-Kerala, T., Finnila, S. & Majamaa, K. Mitochondrial DNA variant m.15218A > G in Finnish epilepsy patients who have maternal relatives with epilepsy, sensorineural hearing impairment or diabetes mellitus. *BMC Med. Genet.* **14**, 73-2350-14-73 (2013).

11. Neuvonen, A. M. *et al.* Vestiges of an Ancient Border in the Contemporary Genetic Diversity of North-Eastern Europe. *PLoS ONE* **10**, 1-19 (2015).
